# Contribution of reactive oxygen species to the pathogenesis of pulmonary arterial hypertension

**DOI:** 10.1371/journal.pone.0180455

**Published:** 2017-06-30

**Authors:** Nikki L. Jernigan, Jay S. Naik, Laura Weise-Cross, Neil D. Detweiler, Lindsay M. Herbert, Tracylyn R. Yellowhair, Thomas C. Resta

**Affiliations:** Vascular Physiology Group, Department of Cell Biology and Physiology, University of New Mexico Health Sciences Center, Albuquerque, NM, United States of America; Vanderbilt University Medical Center, UNITED STATES

## Abstract

Pulmonary arterial hypertension is associated with a decreased antioxidant capacity. However, neither the contribution of reactive oxygen species to pulmonary vasoconstrictor sensitivity, nor the therapeutic efficacy of antioxidant strategies in this setting are known. We hypothesized that reactive oxygen species play a central role in mediating both vasoconstrictor and arterial remodeling components of severe pulmonary arterial hypertension. We examined the effect of the chemical antioxidant, TEMPOL, on right ventricular systolic pressure, vascular remodeling, and enhanced vasoconstrictor reactivity in both chronic hypoxia and hypoxia/SU5416 rat models of pulmonary hypertension. SU5416 is a vascular endothelial growth factor receptor antagonist and the combination of chronic hypoxia/SU5416 produces a model of severe pulmonary arterial hypertension with vascular plexiform lesions/fibrosis that is not present with chronic hypoxia alone. The major findings from this study are: 1) compared to hypoxia alone, hypoxia/SU5416 exposure caused more severe pulmonary hypertension, right ventricular hypertrophy, adventitial lesion formation, and greater vasoconstrictor sensitivity through a superoxide and Rho kinase-dependent Ca^2+^ sensitization mechanism. 2) Chronic hypoxia increased medial muscularization and superoxide levels, however there was no effect of SU5416 to augment these responses. 3) Treatment with TEMPOL decreased right ventricular systolic pressure in both hypoxia and hypoxia/SU5416 groups. 4) This effect of TEMPOL was associated with normalization of vasoconstrictor responses, but not arterial remodeling. Rather, medial hypertrophy and adventitial fibrotic lesion formation were more pronounced following chronic TEMPOL treatment in hypoxia/SU5416 rats. Our findings support a major role for reactive oxygen species in mediating enhanced vasoconstrictor reactivity and pulmonary hypertension in both chronic hypoxia and hypoxia/SU5416 rat models, despite a paradoxical effect of antioxidant therapy to exacerbate arterial remodeling in animals with severe pulmonary arterial hypertension in the hypoxia/SU5416 model.

## Introduction

Pulmonary hypertension is a severe progressive disorder characterized by vasoconstriction and vascular remodeling leading to an increase in pulmonary vascular resistance and cor pulmonale. A variety of conditions can lead to the development of pulmonary hypertension and these have been classified by the World Health Organization into 5 groups based on similarities in pathophysiological mechanisms, clinical manifestation, and therapeutic approaches. Pulmonary hypertension produced by chronic hypoxia (Group III) is largely due to mechanisms of vasoconstriction with moderate medial muscularization of proximal and distal pulmonary arteries. Chronic hypoxia alone causes neither rarefaction [1] nor the obliterative neointimal or other complex vascular lesions that seemingly account for the high pulmonary vascular resistance in human severe pulmonary arterial hypertension (PAH) (Group I). However, chronic hypoxia exposure plus a single injection of the vascular endothelial growth factor receptor antagonist, SU5416, results in a model of severe PAH accompanied by formation of obstructive cellular lesions in the lumen of small pulmonary arteries and arterioles, in addition to increased medial muscularization of proximal and distal pulmonary arteries [2].

A number of studies show increased oxidative stress and decreased antioxidant activity contribute to the enhanced pulmonary vasoconstriction, lung vascular remodeling, and right heart failure associated with pulmonary hypertension [reviewed in [3–7]]. Our laboratory has previously identified a central role for reactive oxygen species in coupling both receptor stimulation and membrane depolarization to RhoA/Rho kinase (ROK) activation and enhanced vasoconstriction in small pulmonary arteries from chronically hypoxic rats [8, 9]. In addition to increased superoxide generation, various animal models of pulmonary hypertension and patients with idiopathic PAH exhibit decreased antioxidant capacity, including a reduction in superoxide dismutase (SOD) expression and activity [10–18]. However, whether an oxidant/antioxidant imbalance contributes to development of severe PAH is unknown. We tested this possibility by comparing effects of the chemical antioxidant, TEMPOL, on the development of pulmonary hypertension, vascular remodeling, and enhanced vasoconstrictor reactivity in rat models of Group I (hypoxia/SU5416) and Group III (chronic hypoxia alone) pulmonary hypertension.

## Materials and methods

All protocols used in this study were reviewed and approved by the Institutional Animal Care and Use Committee of the University of New Mexico School of Medicine.

### Hypoxia/SU5416 rat model

Adult male Sprague-Dawley rats (200–250 g body wt, Harlan Industries) were injected subcutaneously with SU5416 (20 mg/kg) or vehicle, CMC [0.5% (w/v) carboxymethylcellulose sodium, 0.9% (w/v) NaCl, 0.4% (v/v) polysorbate 80, 0.9% (v/v) benzyl alcohol in deionized water]. Animals designated for exposure to chronic hypoxia were housed in a hypobaric chamber with barometric pressure maintained at ∼380 mmHg for 3 wk. The chamber was opened three times per week to provide animals with fresh food, water, and clean bedding. Age-matched control (normoxia) animals were housed at ambient barometric pressure (∼630 mmHg in Albuquerque, NM). All animals were maintained on a 12:12-h light-dark cycle.

To determine the role of ROS in the development of pulmonary hypertension, some animals were administered 4-hydroxy-2,2,6,6-tetramethylpiperidin-1-oxyl (TEMPOL; 1 mM) in the drinking water 5 days prior to and during the 3 wk normoxia or hypoxia/SU5416 exposure. TEMPOL has been shown to dismutate superoxide catalytically, facilitate hydrogen peroxide metabolism by catalase-like actions, and limit formation of toxic hydroxyl radicals produced by Fenton reactions [19].

### Assessment of pulmonary arterial hypertension

Right Ventricular Systolic Pressure (RVSP) was measured as an index of pulmonary arterial systolic pressure in anesthetized rats (2% isoflurane and 98% O_2_ gas mixture). The right jugular vein was cannulated with a saline-filled [0.9% saline and 1,000 U/ml heparin] micro-renathane catheter via an incision in the ventral surface of the neck. The curved catheter was advanced into the right ventricle and pressure was measured via an APT300 pressure transducer with a TAM-A bridge amplifier (Harvard Apparatus). All data were recorded, and heart rate was calculated with a computer-based data-acquisition system (LabChart).

### Determination of right ventricular hypertrophy and polycythemia

Fulton's index, expressed as the ratio of RV weight to left ventricular plus septal (LV + S) weight, was used to assess RV hypertrophy. To examine ventricular structure and assess fibrosis, heart sections were prepared for Heidenhain’s AZAN trichrome stain from paraffin-embedded heart tissue. Hematocrit was measured from blood samples collected in microcapillary tubes after direct cardiac puncture at the time of lung isolation.

### Evaluation of pulmonary arterial remodeling

Lung sections were prepared for hematoxylin and eosin (H&E) stain, Heidenhain’s Azan trichrome stain, and immunofluorescence from paraffin-embedded lung tissue. For immunofluorescence, sections were incubated with the following: anti-smooth muscle α-actin (SMA; 1:600; Abcam, Cambridge, MA, USA), anti-von Willebrand factor (1:250; Bio-Rad, Hercules, CA, USA), anti-vimentin (1:300; Abcam), anti-HSP-47 (1:250, Abcam) and SYTOX (1:10,000; Thermo Fisher, Waltham, MA USA). To evaluate percent muscularization, pulmonary arteries were identified based on morphology and the presence of both smooth muscle α-actin and von Willebrand factor. Images of pulmonary arteries were collected using a 63x objective on a confocal microscope (TCS SP5, Leica) and thresholded using MetaMorph Imaging software (Molecular Devices). Regions of interest (ROIs) were drawn around each fully muscularized artery and the percent thresholded area to total ROI area was calculated for each artery as the percent muscularization, as previously described [20]. Arterial diameter was calculated based on the circumference of the ROI. To determine area of SMA, vimentin, and HSP-47 immunofluorescence, 10 random 20x images were acquired from each lung section and averaged (n = 4 animals/group). Each image was thresholded using MetaMorph Imaging software (Molecular Devices) to select positively-stained areas with fluorescence intensity values above background and analyzed blindly.

### Generation of primary PASMC culture

Rats were anesthetized with pentobarbital sodium (200 mg/kg ip), and the heart and lungs were removed by midline thoracotomy. Distal intrapulmonary arteries (∼4^th^–5^th^ order) were dissected from surrounding *lung parenchyma* and enzymatically digested in reduced-Ca^2+^ Hank’s Balanced Salt Solution (HBSS) containing papain (26 U/ml), type-I collagenase (1750 U/ml), dithiothreitol (1 mg/ml), and BSA (2 mg/ml) at 37°C for 30 min. Single smooth muscle cells were dispersed by gentle trituration with a fire-polished pipette in Ca^2+^-free HBSS. The cell suspension was plated in Ham's F-12 media supplemented with 5% fetal bovine serum and 1% penicillin/streptomycin for 3–4 days in a humidified atmosphere of 5% CO_2_-95% air at 37°C. Cellular purity was >90%, as assessed by morphological appearance and immunofluorescence of anti-smooth muscle 22 alpha (SM-22α).

### Measurement of PASMC superoxide and hydrogen peroxide levels

To determine the effect of hypoxia and hypoxia/SU5416 treatment on O_2_^·−^ levels, PASMC were isolated from each rat as above and incubated with dihydroethidium (DHE; 5 μM in 0.05% pluronic acid) and TO-PRO-3 (1:2,000, Molecular Probes) at 37°C for 15 min. Cells were subsequently fixed with 2% paraformaldehyde for 10 min at room temperature. Fluorescence images were acquired with a 63x objective on a confocal microscope (Leica, TCS SP5). Mean fluorescence intensity was taken per field from 10 images and was averaged from 5 animals per group. Each image was thresholded using MetaMorph Imaging software (Molecular Devices) to select for positively-stained areas with fluorescence intensity values above background (i.e., of cells not treated with DHE). Fluorescence images were digitally inverted to provide better contrast and visibility of immunofluorescence. To confirm the specificity of DHE flourescence in control experiments, PASMC were pre-incubated with either increasing concentrations of H_2_O_2_ (0.3–30 μM) or di-(4-carboxybenzyl) hyponitrite (SOTS-1; 10 μM; Cayman Chemicals). SOTS-1 is an azo-compound that can be thermally decomposed in aqueous solution to generate O_2_^·−^ at physiological pH and exhibits a half-life of ∼90 min [21].

The effect of TEMPOL on PASMC H_2_O_2_ levels was determined by an Amplex Red Hydrogen Peroxide/Peroxidase Assay according to the manufacturer's protocol (Life Technologies). PASMC were pretreated for 30 min with PEG-catalase (250 U/ml) or TEMPOL (1 mM) and incubated with Amplex Red reagent for 30 min at 37°C. The supernatant was transferred to a 96-well plate, and Amplex Red fluorescence was measured with a fluorescence microplate reader (Tecan Infinite M200) using excitation at 550 nm and fluorescence detection at 590 nm. Protein concentration was determined for each well following the assay to normalize H_2_O_2_ production per well.

### Evaluation of H_2_O_2_ induced oxidative stress

Levels of sulfenylated and 4-hydroynonenal (HNE)-modified proteins were measured by Western blotting in lungs from each group of rats as indices of cysteine thiol oxidation and lipid peroxidation, respectively. Lungs were snap-frozen in liquid nitrogen, and stored at -80°C. Whole lung tissue was homogenized in 10 mM Tris·HCl containing 255 mM sucrose, 2 mM EDTA, protease inhibitor cocktail (Cytoskeleton Inc) and phosphatase inhibitor (Sigma-Aldrich) and centrifuged at 1500 g for 10 min to remove insoluble debris. Supernatant was collected, and sample protein concentrations were determined by spectrophotometry (Nano Drop 2000, Thermo Scientific). For measurements of sulfenylated proteins, whole lung lysates were first incubated with 10 mM dimedone (Sigma-Aldrich) on ice for 2 hr prior to preparation for Western blotting, as described by others [22]. Sample buffer without 2-mercaptoethanol was also used to prevent thiol modification. Equal protein concentrations of lung lysates were separated by SDS-PAGE (Tris-HCl gels, Bio-Rad) and transferred to polyvinylidene difluoride membranes. Blots were blocked in TBST + 5% milk + 0.1% sodium azide at RT for 1 hr. Blots were incubated blocking buffer with either 1) rabbit polyclonal 4-hydroxynonenal (HNE) antibody (Abcam, ab46545, 1:1000) overnight at 4°C to detect HNE-modified proteins indicative of lipid peroxidation or 2) rabbit polyclonal antibody against sulfenic acid modified cysteine (Millipore, ABS30, 1:4000) for 1 hr at RT to detect H_2_O_2_ post-translational protein sulfenylation. Lane intensities from 20 kDa– 150 kDa were quantified using ImageJ and normalized to Coomassie staining in the same region. Comparisons between blots were achieved by normalization to an identical control sample present on all blots.

### Cannulation and pressurization of small pulmonary arteries for assessment of vasoreactivity

Rats were anesthetized with pentobarbital sodium (200 mg/kg ip), and the heart and lungs were exposed by midline thoracotomy. The left lung was removed and immediately placed in physiological saline solution (PSS, pH 7.4) containing (in mM) 129.8 NaCl, 5.4 KCl, 0.5 NaH_2_PO_4_, 0.83 MgSO_4_, 19 NaHCO_3_, 1.8 CaCl_2_, and 5.5 glucose (all from Sigma). Fourth- to fifth-order intrapulmonary arteries [100–200 μm inner diameter (ID)] of ∼1-mm length and without visible side branches were dissected free and transferred to a vessel chamber (CH-1, Living Systems). The proximal end of the artery was cannulated with a tapered glass pipette, secured in place with a single strand of silk ligature, and gently flushed to remove any blood from the lumen. The vessel lumen was rubbed with a strand of moose mane to disrupt the endothelium, before securing the distal end of the artery. The vessel was stretched longitudinally to approximate in situ length and pressurized with a servo-controlled peristaltic pump (Living Systems) to 12 mmHg. Arteries were required to hold a steady pressure on switching off the servo-control function to verify the absence of leaks; any vessel with apparent leaks was discarded. The vessel chamber was transferred to the stage of a Nikon Eclipse TS100 microscope where vessels were superfused with PSS equilibrated with a gas mixture containing 10% O_2_, 6% CO_2_, and balance N_2_. A vessel chamber cover was positioned to permit this same gas mixture to flow over the top of the chamber bath. Previous studies from our laboratory [23] indicate that this gas mixture yields an approximate superfusate pH = 7.40, Po2 = 57 mmHg, and Pco2 = 31 mmHg. Bright-field images were obtained with an IonOptix CCD100M camera, and dimensional analysis was performed by IonOptix SarcLen software to measure ID.

### Vessel Ca^2+^ permeabilization to assess endothelin-1-mediated Ca^2+^ sensitization

The effect of hypoxia/SU5416 exposure on endothelin-1 (ET-1; 10^−10^ to 10^−7^ M)-mediated Ca^2+^sensitization was assessed in endothelium-denuded, small pulmonary arteries from vehicle- and SU5416-treated rats exposed to normoxia or hypoxia. Intracellular Ca^2+^ levels ([Ca^2+^]_*i*_) was clamped by permeabilizing with the Ca^2+^ ionophore, ionomycin, similar to that previously described [8, 9, 24]. First, arteries were equilibrated in Ca^2+^-free PSS containing 3 mM EGTA and 3 μM ionomycin to permeabilize the vessels to Ca^2+^. Arteries were then equilibrated in PSS with a calculated free Ca^2+^ concentration of 200 nM [containing (in mM) 129.8 NaCl, 5.4 KCl, 0.5 NaH_2_PO_4_, 1.3 MgSO_4_, 19 NaHCO_3_, 6.8 CaCl_2_, 5.5 glucose, 8.2 EGTA, 0.003 ionomycin (Sigma)]. This free [Ca^2+^] was calculated using the *K*_d_ of EGTA for Ca^2+^ of 43.7 nM and the *K*_d_ of EGTA for Mg^2+^ of 3.33 mM at 37°C and pH 7.4 [25]. This free [Ca^2+^]_i_ produced optimal vasoconstrictor responsiveness to ET-1 with minimal effects on basal tone in Ca^2+^-permeabilized arteries in preliminary experiments. In separate sets of experiments, we assessed the mechanism of Ca^2+^ sensitization by examining the effects of ET-1 responsiveness in the presence of the ROS scavenger, TEMPOL (1 mM) or the ROK inhibitor, HA-1077 (10 μM, Calbiochem).

### Calculations and statistics

All data are expressed as means ± SE. *n* values of numbers of animals in each group. A two- or three-way ANOVA was used to make comparisons when appropriate (SigmaPlot 12.5, Systat Software, Inc). If differences were detected by ANOVA, individual groups were compared with the Student-Newman-Keuls test. *P* values of <0.05 were accepted as significant for all comparisons.

## Results

### TEMPOL attenuates the development of pulmonary hypertension

**Fig 1:**
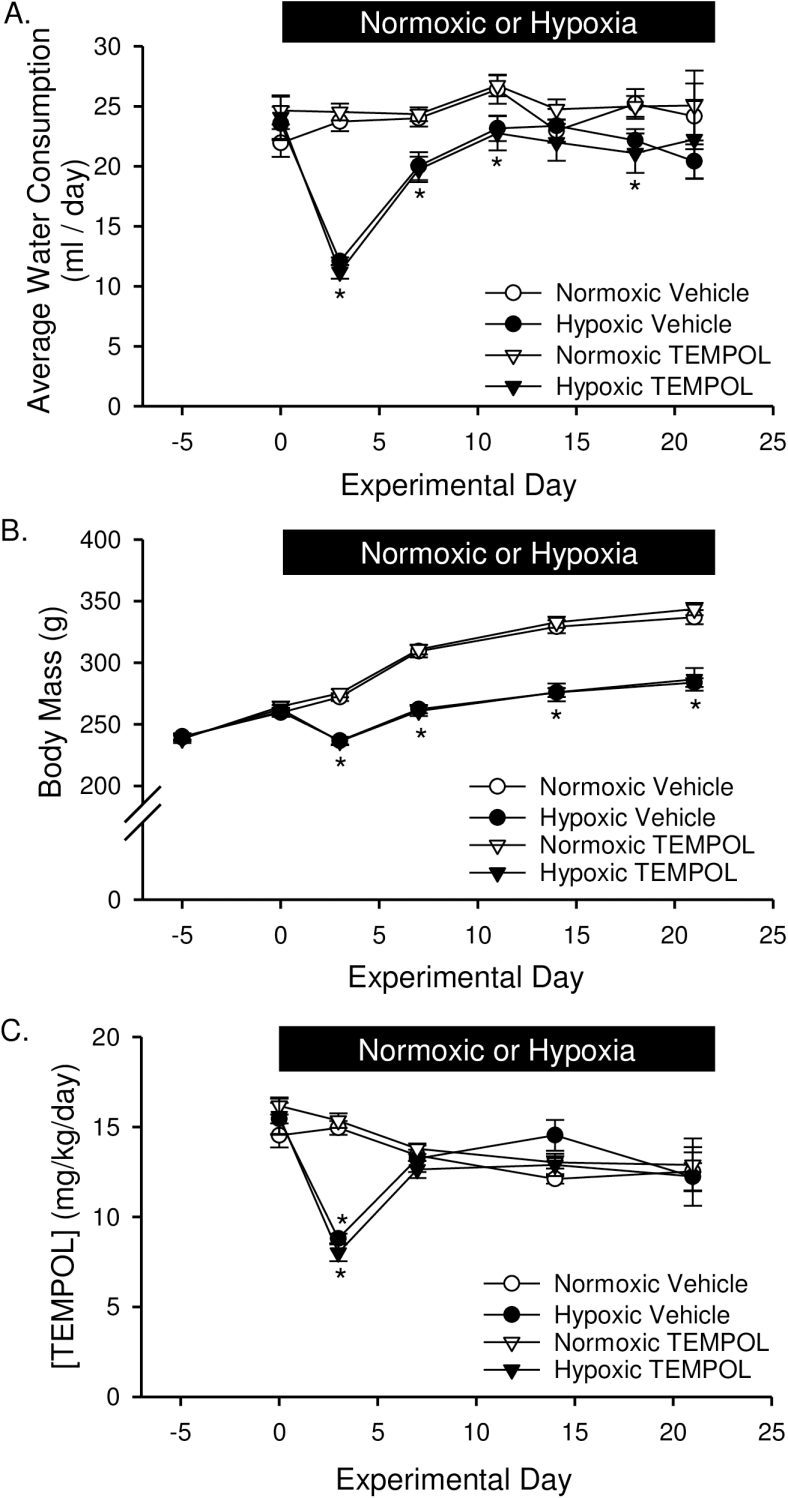
TEMPOL does not effect water consumption or body mass. At the onset of hypoxic exposure, (A) water consumption (ml/day), (B) body mass (g), and (C) concentration of TEMPOL (mg/kg/day) dropped significantly. Water consumption and TEMPOL concentration was largely restored after 1 wk hypoxia. Body mass also increased following 1 wk chronic hypoxia in a parallel fashion to normoxic rats but remained lower due to the initial weight loss. There were no differences between SU5416- and vehicle-treated animals; therefore, data were grouped together for this analysis. Values are means ± SE; n = 10 animals/group. *P < 0.05 hypoxia vs. normoxia; there was no difference between vehicle and TEMPOL treated. Data analyzed with two-way ANOVA and individual groups compared with the Student-Newman-Keuls test.

The administration of TEMPOL was begun 5 days prior to hypoxic exposure and/or SU5416 treatment. TEMPOL alone did not affect the volume of water consumed or body weight (Fig 1). Exposure to hypoxia, however, caused a marked decline in both water consumption (Fig 1A) and body weight (Fig 1B) within the first several days. As a consequence, the delivery rate of TEMPOL (Fig 1C; mg/kg/day) was lower the first couple of days of hypoxic exposure. Water consumption and concentration of TEMPOL returned to normal (Fig 1A and 1C), and although rats gained weight normally after day 3, body mass remained lower throughout hypoxic exposure due to the initial drop.

**Fig 2:**
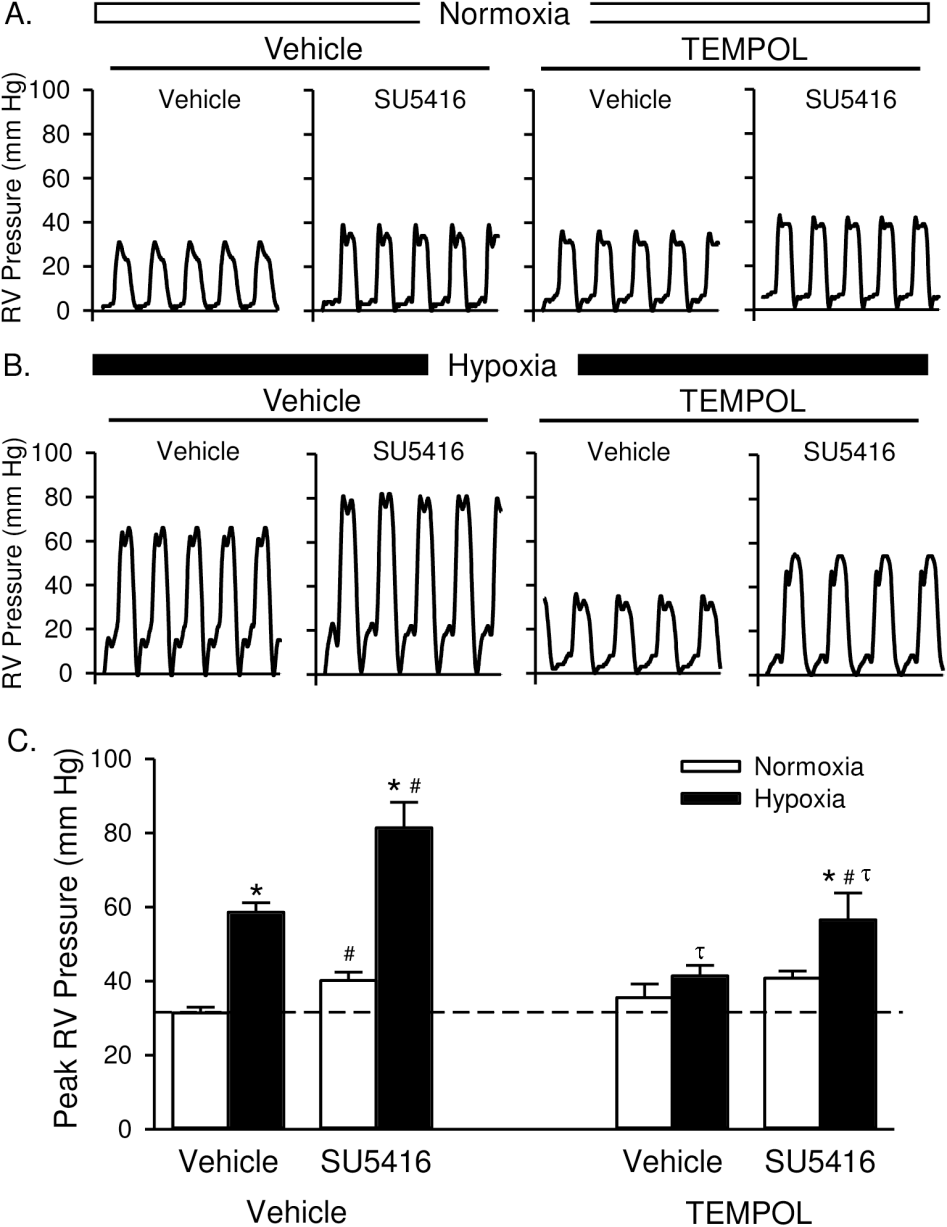
TEMPOL attenuates the development of pulmonary hypertension. Representative traces of right ventricular (RV) pressure measurements (mmHg) in rats treated with vehicle, SU5416, and/or TEMPOL and exposed to normoxia (A) or hypoxia (B). (C) Summary data for peak RVSP (mmHg). Dotted line equals normoxia-vehicle average. Values are means ± SE; n = 5 animals/group. *P < 0.05 vs. the normoxia group; # P < 0.05 vs. the corresponding SU5416 vehicle group; τ p < 0.05 vs. corresponding TEMPOL-vehicle group; analyzed by multiple two-way ANOVA and individual groups compared with the Student-Newman-Keuls test.

Rats treated with SU5416 developed significantly higher RVSP than did vehicle-treated rats under both normoxic and hypoxic conditions (Fig 2). The effect of SU5416 on RVSP in normoxic animals is consistent with initial reports in rats [2], and may be due to the lower barometric pressure (P_B_) in Albuquerque, NM (P_B_ ~ 630 mmHg) versus that at sea-level. TEMPOL did not affect RVSP in normoxic animals but prevented increases in RVSP in animals exposed to hypoxia alone; normalizing responses to those of normoxic animals (Fig 2C). TEMPOL also attenuated hypoxia/SU5416-induced increases in RVSP, although values remained elevated compared to both normoxic controls and TEMPOL-treated hypoxic rats (Fig 2C). Heart rate was not different between groups (Table 1; normoxia vs hypoxia p = 0.66; vehicle vs SU5416 p = 0.34; vehicle vs TEMPOL p = 0.913).

**Table 1:** Body weight, heart weight ratios, hematocrit, and heart rate from rats treated with TEMPOL, SU5416 or vehicle and exposed to normoxia or hypoxia.

	Final BW (g)	RV/BW (mg/g)	LV/BW (mg/g)	RV/Total (mg/mg)	Hematocrit (%)	Heart Rate (beats/min)
**Normoxia Vehicle**						
Vehicle	337 ± 4 (24)	0.61 ± 0.01 (19)	2.23 ± 0.04 (19)	0.21 ± 0.003 (21)	45.6 ± 0.4 (17)	315 ± 12 (5)
TEMPOL	331 ± 4 (11)	0.58 ± 0.02 (5)	2.06 ± 0.05 (5) τ	0.22 ± 0.01 (5)	44.5 ± 1.1 (6)	322 ± 14 (5)
**Normoxia SU5416**						
Vehicle	330 ± 5 (14)	0.75 ± 0.06 (13) ^#^	2.36 ± 0.04 (13) ^#^	0.24 ± 0.01 (13) ^#^	44.3 ± 0.5 (7)	287 ± 16 (5)
TEMPOL	338 ± 6 (11)	0.67 ± 0.03 (5)	2.04 ± 0.05 (5) τ	0.25 ± 0.004 (5) ^#^	43.8 ± 1.3 (6)	301 ± 24 (5)
**Hypoxia Vehicle**						
Vehicle	284 ± 3 (27) *	1.11 ± 0.02 (23) *	2.19 ± 0.03 (23)	0.34 ± 0.005 (23) *	68.4 ± 0.6 (24) *	312 ± 13 (5)
TEMPOL	297 ± 6 (11) *τ	0.99 0.06 (5) *	2.04 ± 0.05 (5)	0.32 ± 0.02 (5) *	69.0 ± 0.6 (6) *	291 ± 9 (5)
**Hypoxia SU5416**						
Vehicle	270 ± 4 (30) *^#^	1.53 ± 0.04 (25) * ^#^	2.30 ± 0.05 (25) * ^#^	0.40 ± 0.008 (26) * ^#^	63.8 ± 0.8 (27) * ^#^	298 ± 15 (5)
TEMPOL	263 ± 3 (11) *^#^	1.58 ± 0.02 (5) * ^#^	2.42 ± 0.06 (5) * ^#^	0.40 ± 0.003 (5) * ^#^	62.5 ± 1.2 (6) * ^#^	297 ± 33 (5)

Values are means ± SE; *n* = animals/group (indicated in parentheses).

*P < 0.05 vs. the corresponding normoxia group

# *P* < 0.05 vs. the corresponding SU5416-vehicle group

τ P < 0.05 vs. the corresponding TEMPOL-vehicle group; analyzed with two- or three-way ANOVA and individual groups compared with the Student-Newman-Keuls test. BW, body weight; RV, right ventricle; LV, left ventricle.

### Right ventricular hypertrophy is not attenuated by TEMPOL

**Fig 3:**
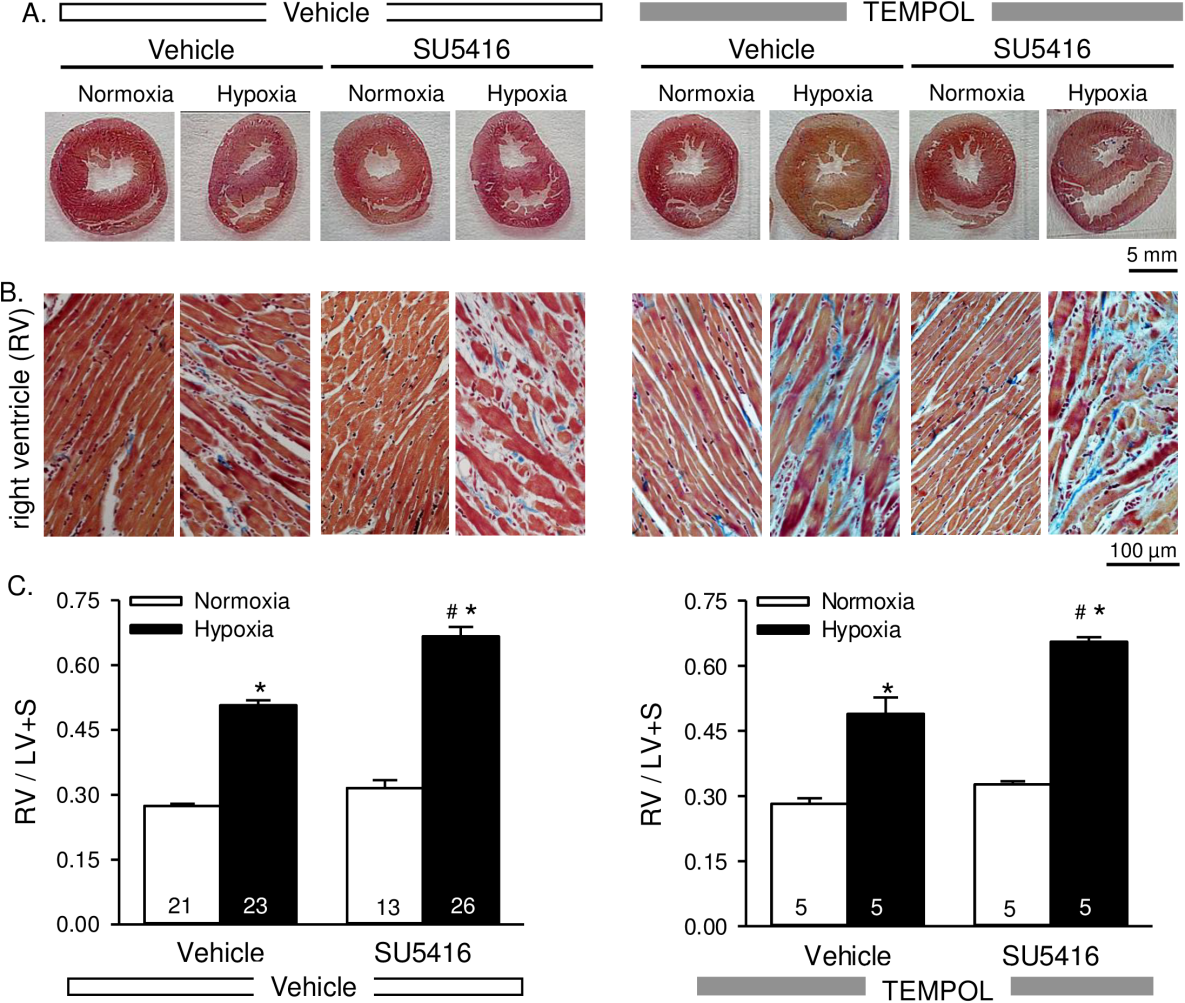
Right ventricular hypertrophy is not attenuated by TEMPOL. Representative AZAN trichrome-stained whole heart sections (A) and higher magnification images of the right ventricle (B) from rats treated with vehicle, SU5416, and/or TEMPOL and exposed to normoxia or hypoxia. AZAN trichrome shows cell nuclei (dark red), collagen (blue) and orange-red in cytoplasm. C) Summary data showing Fulton’s index [ratio of RV to left ventricular plus septal (LV + S) heart weight] Values are means ± SE; n = animals/group (indicated in bars). *P < 0.05 vs. the normoxia group; # P < 0.05 vs. the corresponding SU5416 vehicle group; analyzed with multiple two-way ANOVA and individual groups compared with the Student-Newman-Keuls test.

There were clear morphologic changes to the heart including a thickened right ventricular wall, dilated right ventricle, compressed left ventricle, and flattened septum in animals exposed to hypoxia and hypoxia/SU5416 (Fig 3A). Collagen deposition was also evident in the right ventricular wall following hypoxic exposure that was more pronounced with hypoxia/SU5416 (Fig 3B). Both right ventricular and left ventricular weights were greater in normoxic and hypoxic SU5416-treated rats (Table 1). Although right ventricular to total ventricular weight was greater in normoxic/SU5416 compared to normoxic vehicle-treated rats (Table 1), RV/LV + S was not statistically different (*p* = 0.155; Fig 3C). In addition, there was a statistically significant interaction between SU5416 treatment and hypoxia exposure indicated by the greater RVSP and right ventricular mass in the presence of both insults (Fig 3C; Table 1). As expected, hypoxia was associated with increased hematocrit, however values were significantly less in hypoxia/SU5416 compared to hypoxia alone (Table 1). TEMPOL had no significant effect on heart weights, body weights, RV/LV+S, or hematocrit (Fig 3 and Table 1).

### TEMPOL does not attenuate pulmonary arterial remodeling in response to hypoxia alone, and exacerbates remodeling in rats with severe PAH

**Fig 4:**
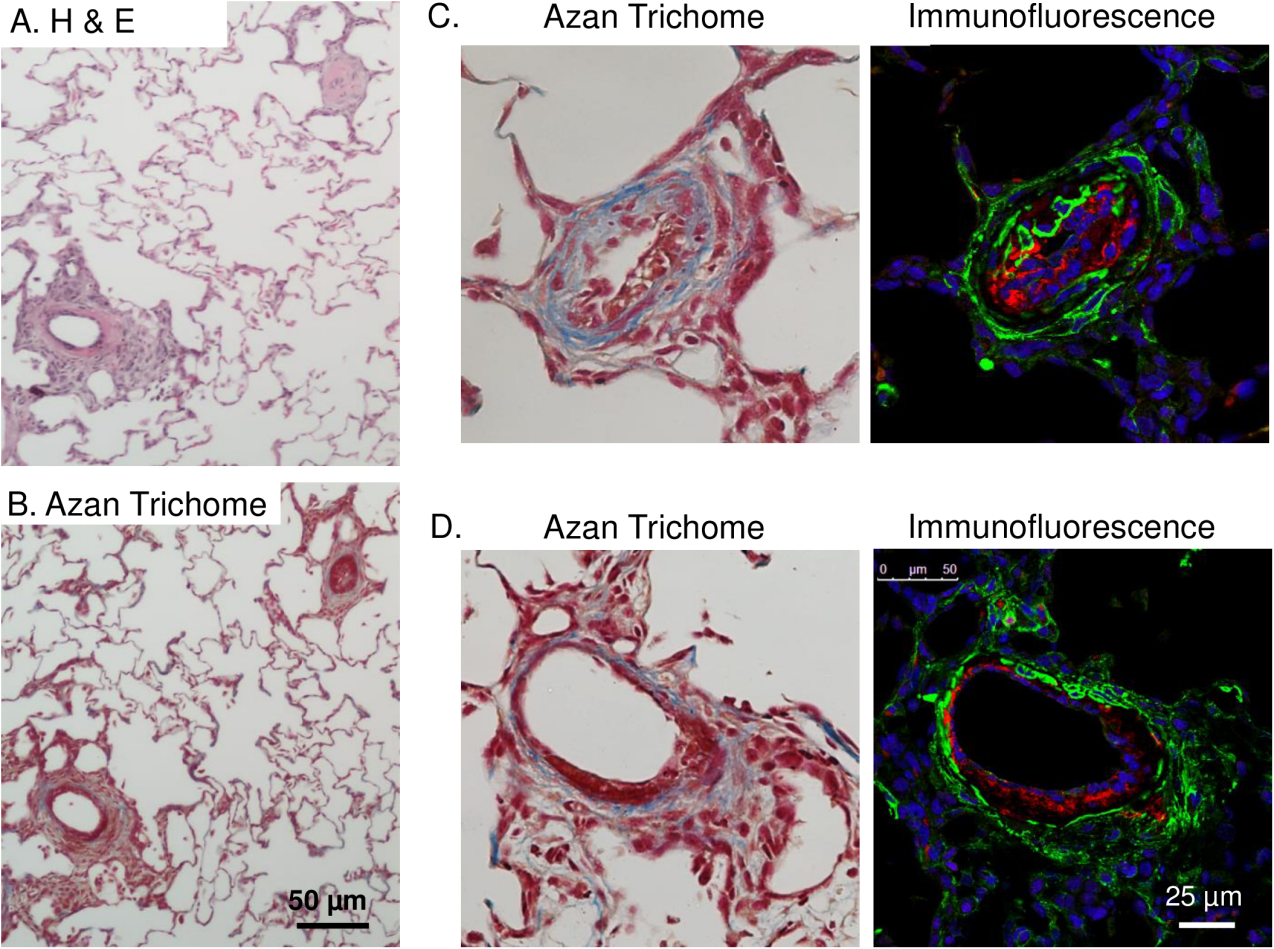
Hypoxia/SU5416 treatment causes both neointimal proliferation of endothelial cells and early plexiform lesions with collagen deposition. All images are from hypoxia/SU5416-treated rats. (A-B) Representative H&E- and Azan trichrome-stained lung sections. (C-D) Higher magnification images of AZAN-stained and fluorescently-labeled arteries showing medial fibrosis and neointimal proliferation of endothelial cells and an early plexiform lesion. AZAN trichrome shows cell nuclei (dark red), collagen (blue) and orange-red in cytoplasm. Fluorescence labeling shows smooth muscle α-actin (green), Von Willebrand factor (red), and sytox (blue).

Hypoxia/SU5416 rats also displayed signs of pulmonary arterial lesions that were not present with hypoxia alone (Fig 4). Staining was performed in two lung sections from each of 4 animals/group (8 groups total). Similar to previous reports [26], we observed two general types of complex lesion formations. Less common (generally 0–2 lesions per lung section), were plexiform-like neointimal lesions demonstrating Von Willebrand factor immunoreactivity combined with medial collagen deposition (Fig 4C). In contrast, the majority of vessels displayed various degrees of hypercellular lesions projecting outward from the medial and adventitial layers often extending into the adjacent lung parenchyma (Fig 4D). The cells within these lesions expressed smooth muscle α-actin (SMA), though the fluorescence intensity was generally lower in these cells compared to those in the medial layer. The medial layer in these lesions also appeared to be fragmented with prominent collagen deposition. Together, these observations suggest the lesions consist largely of myofibroblasts.

**Fig 5:**
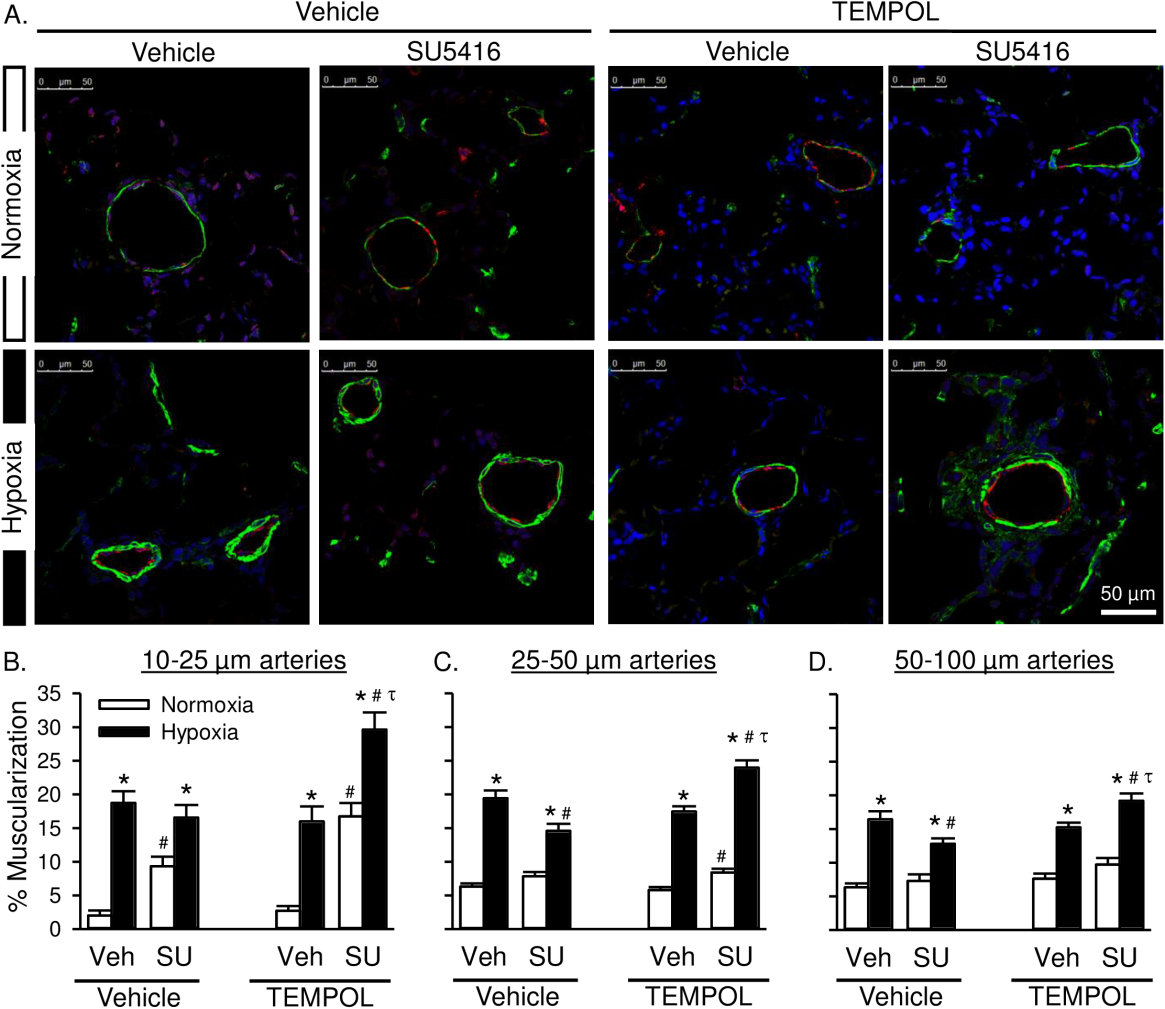
Superoxide scavenging exacerbates arterial remodeling in rats with severe PAH. A) Representative immunofluorescence images of lung sections from normoxic (top row) and hypoxic (bottom row) rats treated with SU5416 and/or TEMPOL. Smooth muscle α-actin (green), Von Willebrand factor (red), and sytox (blue). B) Percent muscularization calculated as percent thresholded smooth muscle α-actin area divided by total arterial wall area according to arterial diameter: 10–25 μm (left), 25–50 μm (middle), and 50–100 μm (right). Values are means ± SE; n = 4 animals/group. *P ≤ 0.05 vs. normoxic group; # P < 0.05 vs. corresponding SU5416-vehicle group; τ p < 0.05 vs. TEMPOL-vehicle group; analyzed by multiple two-way ANOVA and individual groups compared with the Student-Newman-Keuls test.

Hypoxia increased medial hypertrophy of arteries ranging from 10–100 μm in diameter (Fig 5). In normoxic animals, SU5416 increased muscularization of pre-capillary arterioles (10–25 μm; Fig 5B). Hypoxia/SU5416 did not further increase the percent muscularization of arteries compared to animals exposed to hypoxia alone, as assessed by expression of SMA in the medial layer. Rather, the percent muscularization was significantly lower in 25–50 μm and 50–100 μm arteries from hypoxia/SU5416 compared to hypoxia rats (Fig 5C and 5D). In contrast to effects of chronic TEMPOL treatment to limit increases in RVSP in both hypoxia alone and hypoxia-SU5416 rats, TEMPOL was without effect on the arterial remodeling response in the hypoxia alone group, and unexpectedly augmented percent muscularization in hypoxia/SU5416 rats.

**Fig 6:**
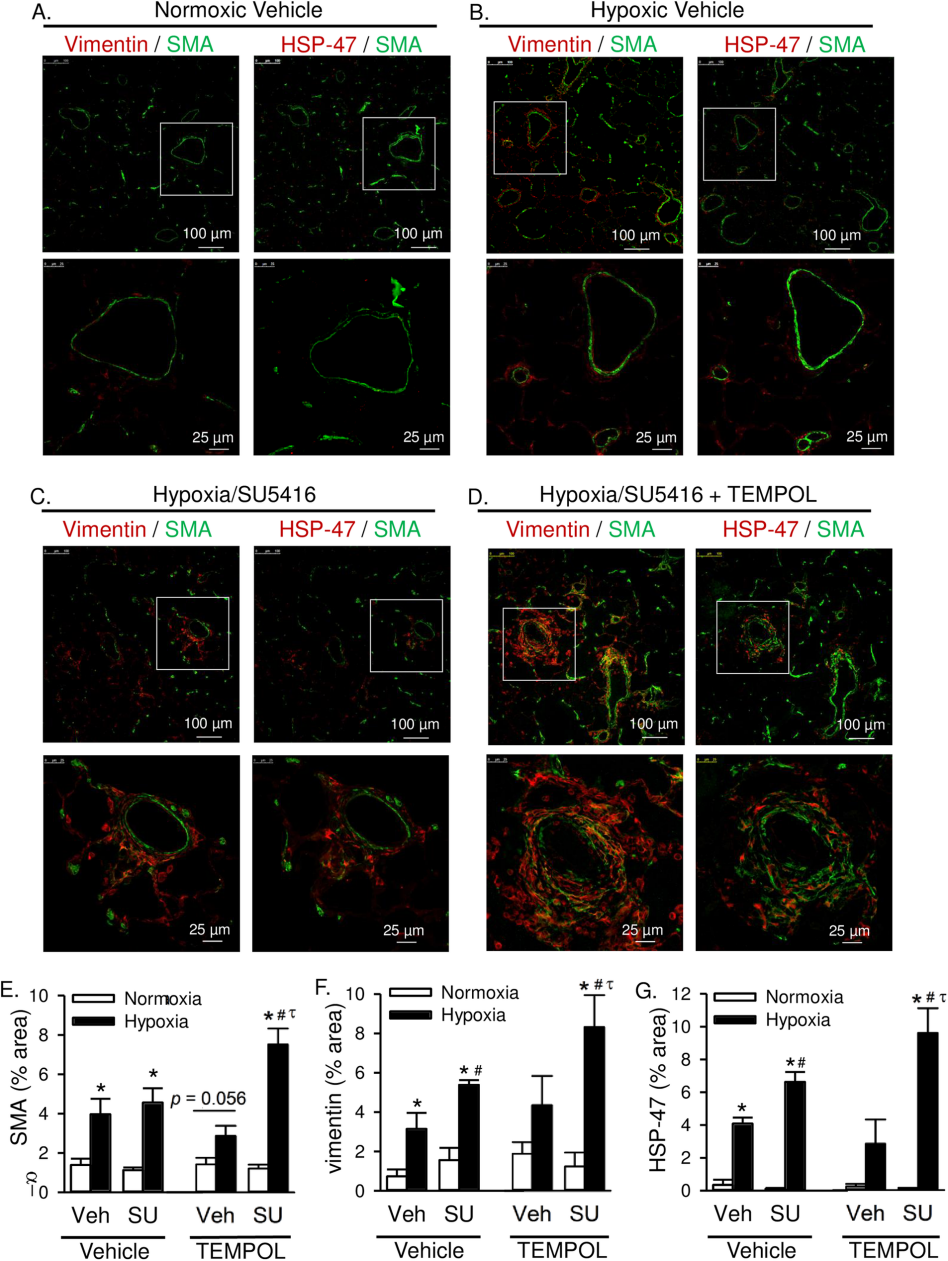
Superoxide scavenging exacerbates adventitial remodeling in rats with severe PAH. Representative immunofluorescence images of lung sections from rats treated with normoxic vehicle (A), hypoxic vehicle (B), hypoxia/SU5416 (C), or hypoxia/SU5416/TEMPOL (D). Sections were incubated with anti-smooth muscle α-actin (SMA, green) and either vimentin (red, left) or HSP-47 (red, right). Summary data showing average percent area of positive immunofluorescence from 10 random 20x images per lung section for E) SMA, F) vimentin, and G) HSP-47. Values are means ± SE; n = 4 animals/group. *P ≤ 0.05 vs. normoxic group; # P < 0.05 vs. corresponding SU5416-vehicle group; τ p < 0.05 vs. TEMPOL-vehicle group; analyzed by multiple two-way ANOVA and individual groups compared with the Student-Newman-Keuls test.

In addition to this effect of TEMPOL on medial hypertrophy, the incidence and size of outward hypercellular lesions (shown in Fig 4D) were greater in TEMPOL-treated hypoxia/SU5416 rats than in vehicle-treated hypoxia/SU5416 rats. As noted earlier, many cells in these fibrotic lesions express SMA, suggestive of myofibroblasts. The area of SMA immunofluorescence in these lung sections was largely increased in TEMPOL-treated hypoxia/SU5416 rats (Fig 6A–6E). This possibility was further examined by co-labeling with the mesenchymal marker vimentin to identify fibroblasts/myofibroblasts [27]. Whereas there was minimal vimentin immunoreactivity in normoxic vehicle lung sections (Fig 6A), there was a progressive increase in perivascular expression of vimentin in hypoxic vehicle (Fig 6B), hypoxia/SU5416 (Fig 6C), and hypoxia/SU5416 with TEMPOL groups (Fig 6D and 6F). Although some cells expressed both SMA and vimentin indicative of myofibroblasts, there was a clear distinction between SMA-positive medial vascular smooth muscle and vimentin-positive fibroblasts.

Heat shock protein 47 (HSP-47) is a collagen specific molecular chaperone involved in the maturation and secretion of procollagen. It is expressed in all collagen-synthesizing cells, such as (myo)fibroblasts, and the expression levels directly correlate with the amounts of collagen being synthesized [28]. Furthermore, there is an excessive accumulation of HSP-47 in several fibrotic diseases [29]. Consistent with collagen deposition in medial and adventitial fibrotic lesions of hypoxia/SU5416 rats (Fig 4), HSP-47 and vimentin localized to similar cellular regions (Fig 6A–6D and 6G), further suggesting (myo)fibroblasts within the hypercellular lesions actively synthesize collagen (seen in Fig 4). TEMPOL had a profound effect in hypoxia/SU5416 treated rats to exacerbate both vimentin and HSP-47 immunofluorescence (Fig 6F and 6G).

### Chronic TEMPOL treatment decreases superoxide levels in pulmonary arterial smooth muscle cells

**Fig 7:**
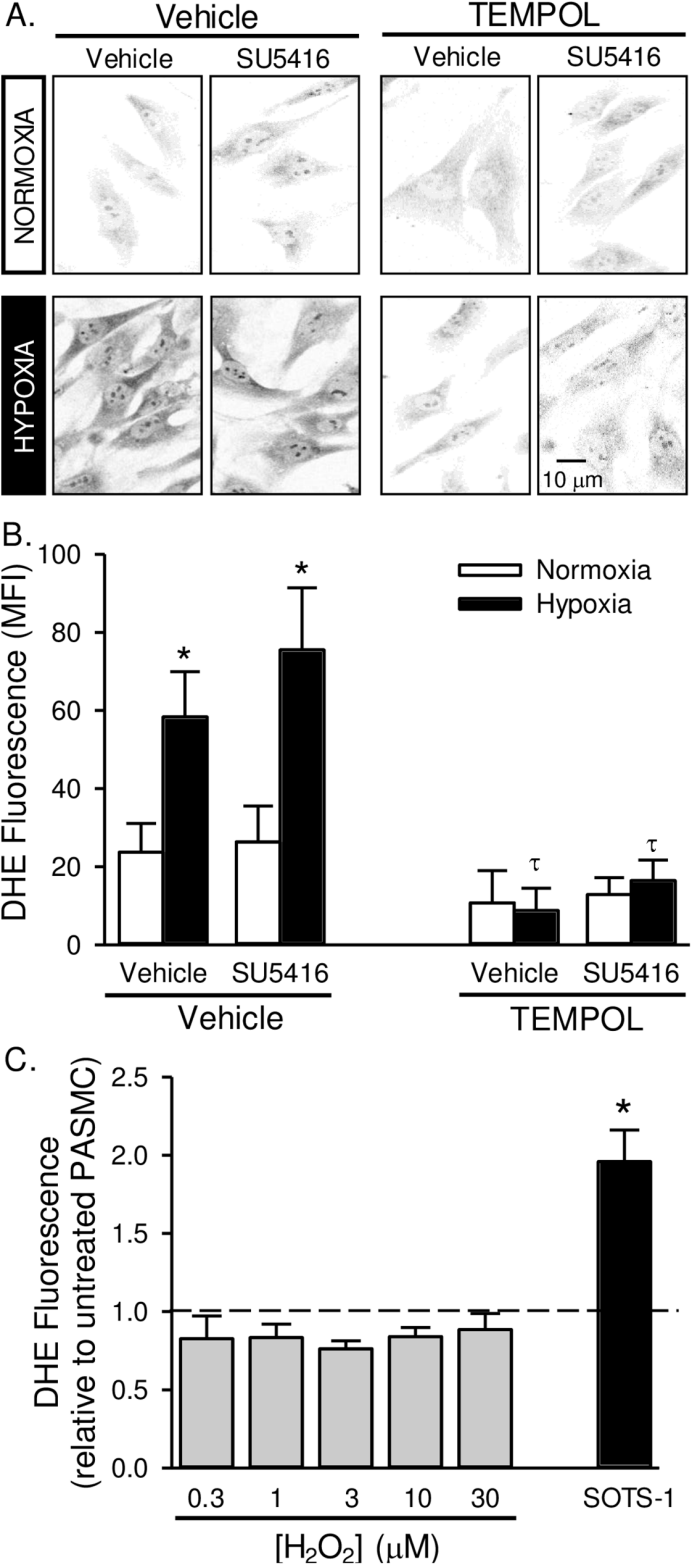
Superoxide levels are increased in pulmonary artery smooth muscle cells from pulmonary hypertensive rats. Representative images (A) and summary data (B) showing background-subtracted mean fluorescence intensity (MFI) of dihydroethidium (DHE) in pulmonary arterial smooth muscle cells from rats treated with SU5416, TEMPOL or vehicle and exposed to normoxia or hypoxia. Fluorescence images were digitally inverted to provide improved signal contrast. Values are means ± SE; n = 5 animals per group; *P < 0.05 vs. corresponding normoxia group; τ p < 0.05 vs. corresponding TEMPOL-vehicle group; analyzed by multiple two-way ANOVA and individual groups compared with the Student-Newman-Keuls test. C) DHE fluorescence in PASMC following pre-incubation with increasing doses of H_2_O_2_ (0.3–30 μM) or SOTS-1 (10 μM). Dotted line represents untreated cells. Values are means ± SE; n = 5 animals per group; *P < 0.05 vs. untreated cells; analyzed by one-way ANOVA.

We next determined the effect of TEMPOL to reduce ROS and oxidative stress. Superoxide was measured in PASMC with DHE. DHE fluorescence was greater in pulmonary arterial smooth muscle cells following hypoxic exposure (Fig 7). SU5416 treatment did not significantly alter DHE fluorescence compared to vehicle-treated rats. Chronic *in vivo* administration of TEMPOL largely decreased DHE fluorescence in cells from hypoxic rats. DHE fluorescence also tended to be reduced by TEMPOL in pulmonary arterial smooth muscle cells from normoxic rats, but this effect did not reach statistical significance (*p* = 0.099; Fig 7).

We previously determined the specificity of DHE to detect O_2_^·−^ over H_2_O_2_ in this preparation by demonstrating that PEG-superoxide dismutase, but not PEG-catalase decreases DHE fluorescence [15]. Additionally, here we show that DHE does not change in PASMC pre-incubated with increasing doses of H_2_O_2_ (Fig 7C). As a control we used SOTS-1 which is an azo-compound that can be thermally decomposed in aqueous solution to generate O_2_^·−^ at physiological pH and exhibits a half-life of ∼90 min [21].

**Fig 8:**
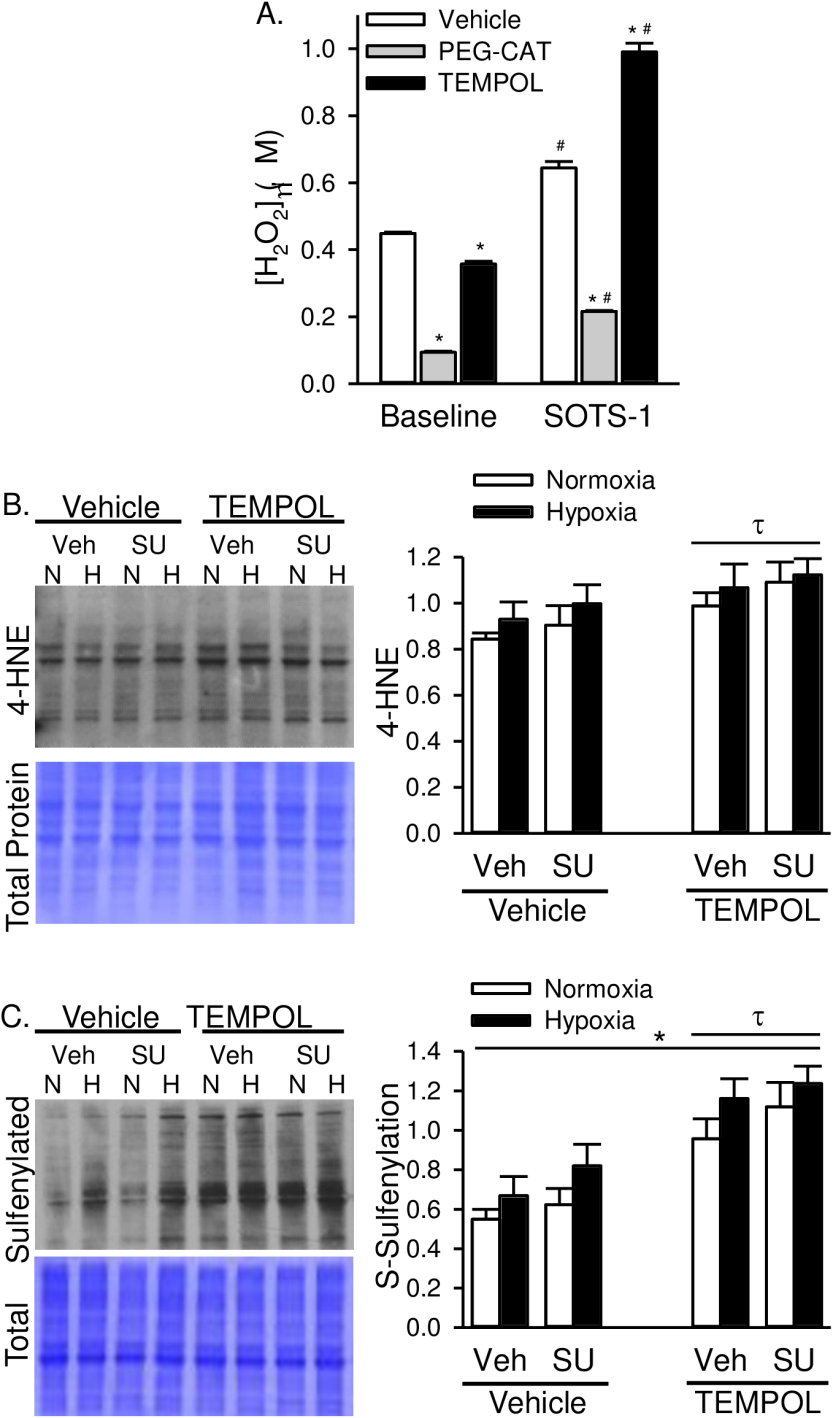
TEMPOL increases H_2_O_2_-specific oxidative stress. A) H_2_O_2_ levels assessed by Amplex Red Assay in control PASMC in the absence or presence of PEG-catalase (PEG-CAT; 250 U/ml) or TEMPOL (1 mM). SOTS-1 (0.01 mM) is a superoxide donor and used to stimulate increased oxidative stress. Values are means ± SE; *n* = 5 animals per group; *P ≤ 0.05 vs. vehicle-treated group; # P < 0.05 vs. baseline; analyzed by two-way ANOVA and individual groups compared with the Student-Newman-Keuls test. Representative western blot and summary data showing B) 4-HNE and C) S-sulfenylated proteins in whole lung homogenates from normoxic and hypoxic rats treated with SU5416 and/or TEMPOL. Values are means ± SE; *n* = 6 animals per group; *P ≤ 0.05 vs. normoxic group; τ p < 0.05 vs. TEMPOL-vehicle group; analyzed by multiple two-way ANOVA and individual groups compared with the Student-Newman-Keuls test.

The antioxidant properties of TEMPOL include both superoxide dismutase (SOD)- and catalase-like activity [19]. Although *in vivo* TEMPOL treatment dramatically reduced PASMC superoxide levels (Fig 7B), we also wanted to determine the effect of acute administration of TEMPOL on PASMC H_2_O_2_ production, and chronic *in vivo* TEMPOL treatment on H_2_O_2_-mediated oxidative stress. Similar to our previous data [15], we show that the release of O_2_^·−^ by SOTS-1- subsequently increases H_2_O_2_ levels in PASMC from control rats as measured by Amplex Red (Fig 8A). PEG-catalase reduced H_2_O_2_ levels under baseline conditions and following SOTS-1 administration (Fig 8A). TEMPOL had a small but significant effect to reduce H_2_O_2_ levels under baseline conditions, however in the presence of excess SOTS-1-derived O_2_^·−^, TEMPOL caused an increase in Amplex Red fluorescence. These data indicate that TEMPOL can act as an SOD mimetic and likely has greater SOD- than catalase-like activity.

Next, we examined the effect of *in vivo* TEMPOL-treatment on H_2_O_2_-induced oxidative stress. We examined levels of 4-hydroxynonenal (4-HNE) as a measure of lipid peroxidation and protein sulfenylation as a measure of H_2_O_2_ post-translational protein modification in whole lung homogenates. Animals treated with TEMPOL demonstrated increased 4-HNE and sulfenylated proteins (Fig 8B and 8C). Hypoxia alone also resulted in increased sulfenylated proteins, however there was no significant interaction between TEMPOL treatment and hypoxia or SU5416. These data demonstrate an effect of TEMPOL to increase H_2_O_2_-induced oxidative stress, however this response does not appear to be exaggerated by hypoxia or SU5416.

### ROS/Rho kinase mediate enhanced endothelin-1 induced Ca^2+^ sensitization and vasoconstriction

**Fig 9:**
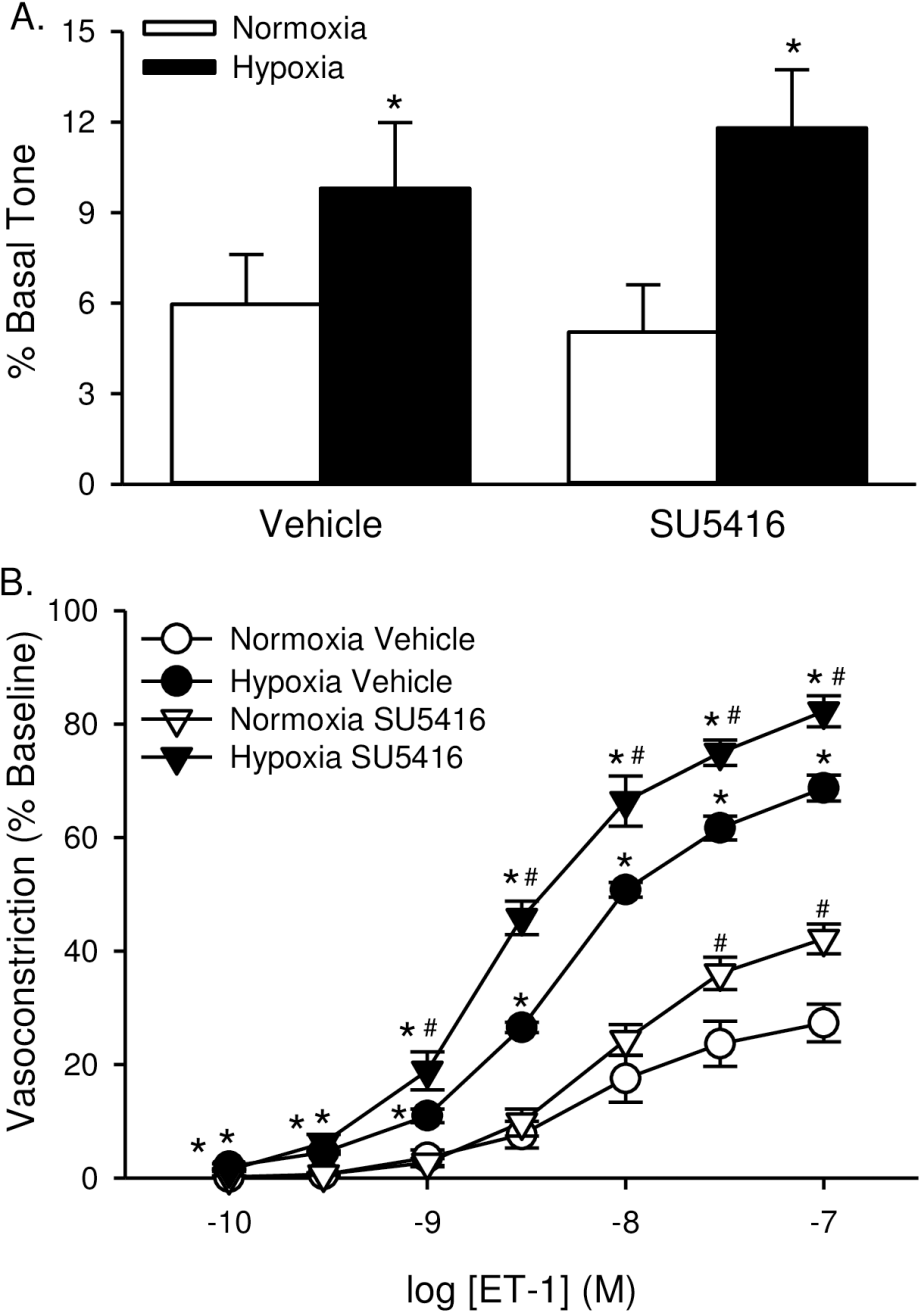
ET-1-induced VSM Ca^2+^ sensitization and vasoconstriction are augmented in small pulmonary arteries from animals treated with SU5614. (A) Basal tone (baseline diameter as a % of Ca^2+^-free diameter) in non-permeabilized, endothelium-disrupted, pressurized small pulmonary arteries. (B) vasoconstriction (% baseline diameter) to endothelin-1 (ET-1; 10^−10^ to 10^−7^ M) in Ca^2+^-permeabilized, endothelium-disrupted, pressurized small pulmonary arteries from rats treated with vehicle or SU5416 and exposed to normoxia or hypoxia. Values are means ± SE; n = 5–6 animals/group. *P ≤ 0.05 vs. normoxic group; #P <0.05 vs. corresponding vehicle treatment; analyzed by two-way ANOVA and individual groups compared with the Student-Newman-Keuls test.

We previously reported that chronic hypoxia alone induces spontaneous tone in small pulmonary arteries and augments vasoconstrictor reactivity to ET-1 through RhoA-dependent myofilament Ca^2+^ sensitization [9, 30]. Consistent with these findings, baseline vascular tone was greater in non-permeabilized arteries from animals exposed to hypoxia compared to normoxia (Fig 9A). SU5416 did not further increase basal tone. Hypoxia also increased vasoconstrictor reactivity in response to ET-1 in Ca^2+^-permeabilized arteries (Fig 9B). SU5416 further augmented ET-1-induced vasoconstriction in arteries from both normoxic and hypoxic rats.

**Fig 10:**
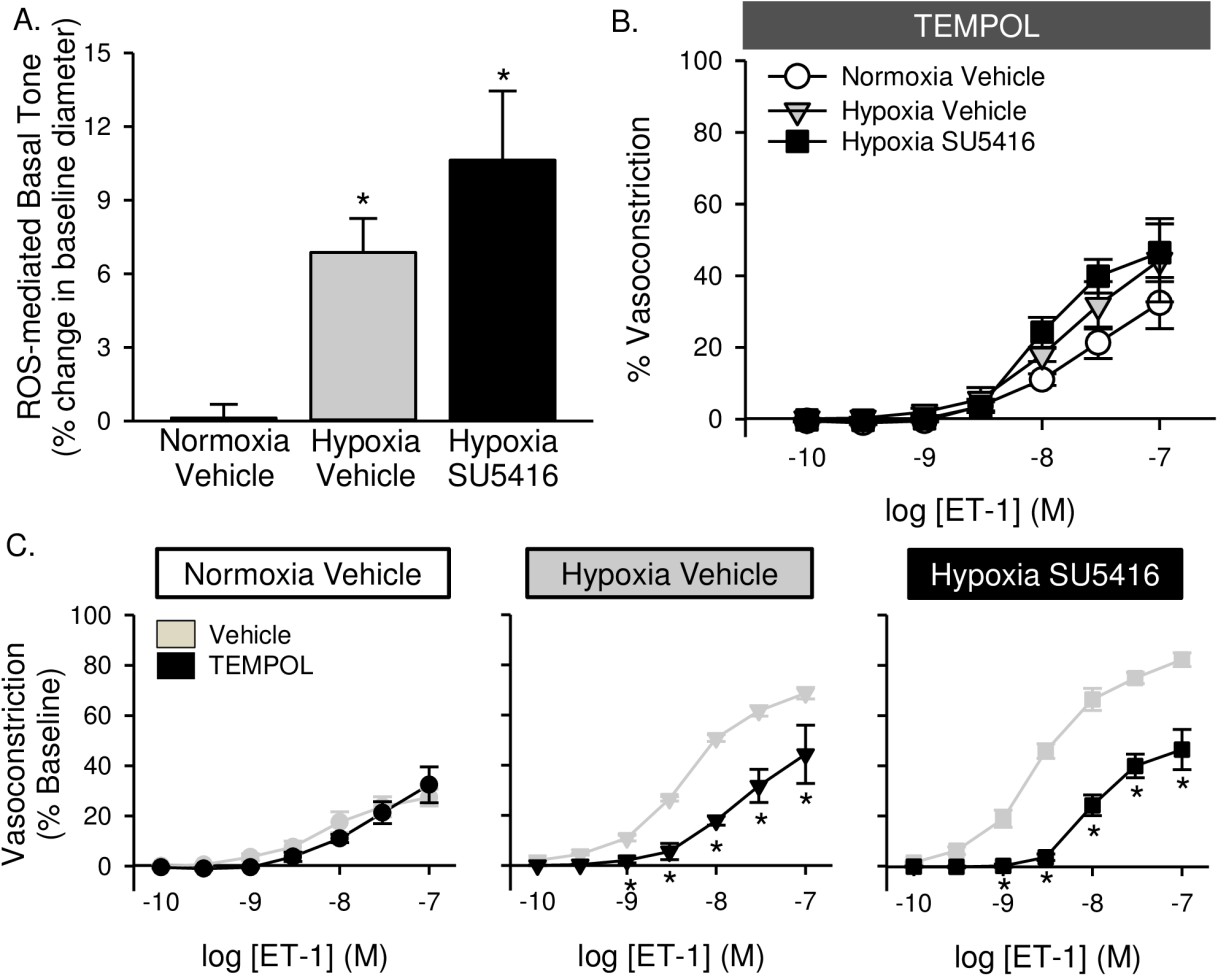
Enhanced basal tone and ET-1-induced pulmonary VSM Ca^2+^ sensitization following hypoxia/SU5416 is mediated by reactive oxygen species. (A) TEMPOL-mediated reversal of basal tone (% baseline diameter) in non-permeabilized, endothelium-disrupted, pressurized small pulmonary arteries. (B) Vasoconstriction (% baseline diameter) to endothelin-1 (ET-1; 10^−10^ to 10^−7^ M) in the presence of TEMPOL (1 mM) in Ca^2+^-permeabilized, endothelium-disrupted, pressurized small pulmonary arteries from normoxic and hypoxic rats treated with vehicle or SU5416. (C) Effect of TEMPOL on ET-1 mediated vasoconstriction in each group compared to vehicle-treated arteries (from Fig 9). Values are means ± SE; n = 4–5 animals/group; * p < 0.05 vs. vehicle-treated arteries; analyzed by two-way ANOVA and individual groups compared with the Student-Newman-Keuls test.

**Fig 11:**
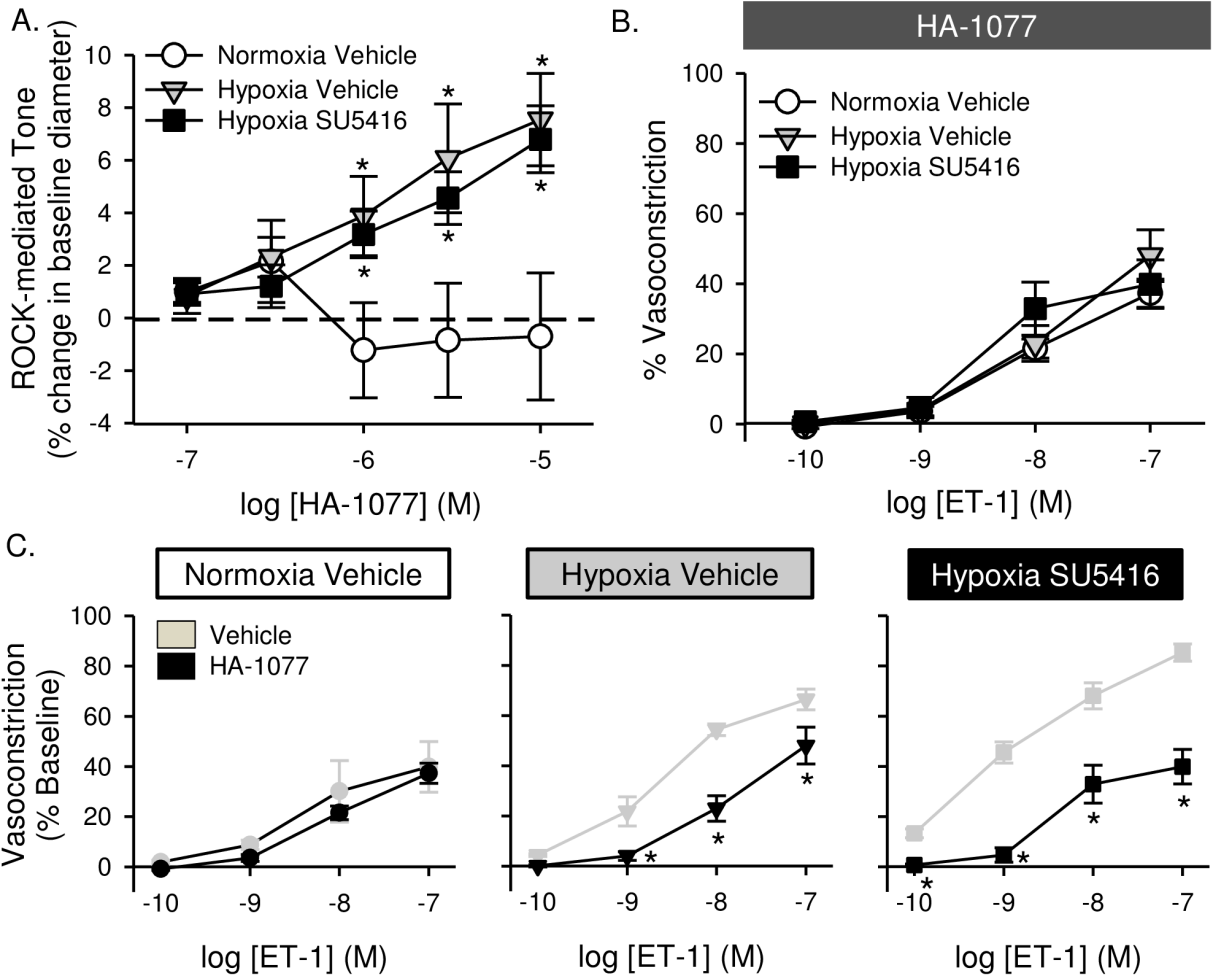
Enhanced basal tone and ET-1-induced pulmonary VSM Ca^2+^ sensitization following hypoxia/SU5416 is mediated by Rho kinase (ROK). (A) HA-1077-mediated change in vessel diameter (% baseline diameter) in non-permeabilized, endothelium-disrupted, pressurized small pulmonary arteries. (B) Vasoconstriction (% baseline diameter) to endothelin-1 (ET-1; 10^−10^ to 10^−7^ M) in the presence of HA-1077 (10 μM) in Ca^2+^-permeabilized, endothelium-disrupted, pressurized small pulmonary arteries from normoxic and hypoxic rats treated with vehicle or SU5416. (C) Effect of HA-1077 on ET-1 mediated vasoconstriction in each group compared to vehicle-treated arteries (from Fig 9). Values are means ± SE; n = 4–5 animals/group; * p < 0.05 vs. vehicle-treated arteries; analyzed by two-way ANOVA and individual groups compared with the Student-Newman-Keuls test.

Acute administration of either TEMPOL (Fig 10A) or the ROK inhibitor HA-1077 (Fig 11A) caused a reduction in baseline vascular tone, suggesting that basal tone following hypoxic exposure (seen in Fig 9) is largely mediated by ROS and ROK. Furthermore, both TEMPOL and HA-1077 significantly diminished ET-1-mediated vasoconstriction in arteries from hypoxia and hypoxia/SU5416 animals (Figs 10C and 11C), effectively normalizing responses between groups (Figs 10B and 11B). Vasoreactivity to ET-1 was unaltered by either inhibitor in vessels from normoxic rats (Figs 10C and 11C).

## Discussion

The present study examined the contribution of ROS to increased RVSP, pulmonary arterial remodeling, and enhanced vasoconstrictor reactivity in rat models of Group III (hypoxia alone) and Group I (hypoxia/SU5416) pulmonary hypertension. The major findings from this study are: 1) severe PAH in hypoxia/SU5416 rats was associated with greater adventitial lesion formation and enhanced vasoconstrictor reactivity to ET-1 through a ROS and ROK-dependent Ca^2+^ sensitization mechanism compared to chronic hypoxia alone; 2) in contrast, SU5416 did not potentiate either chronic hypoxia-induced medial muscularization or ROS generation in pulmonary arterial smooth muscle cells; 3) despite a dramatic effect of the antioxidant, TEMPOL, to limit vasoconstrictor responsiveness and increases in RVSP in each rat model, we observed a paradoxical effect of TEMPOL to exacerbate both medial and adventitial remodeling in animals with severe PAH. Furthermore, TEMPOL had no effect to reduce RV hypertrophy. Together, these studies support a major role for ROS in mediating the vasoconstrictor component of PAH, however there may be therapeutic limitations of using TEMPOL in severe PAH due to exacerbation of medial remodeling and adventitial lesion formation.

Pulmonary hypertension is typically characterized by vascular remodeling and increased production of vasoconstrictors (e.g. ROS and endothelin-1), elevated pulmonary arterial smooth muscle cell [Ca^2+^]_i_ levels, and enhanced sensitivity of the contractile apparatus to Ca^2+^. Activation of the RhoA/ROK pathway and consequent inhibition of myosin light chain phosphatase in pulmonary arterial smooth muscle cell plays an important role in mediating the vasoconstriction associated with chronic hypoxia [8, 9, 31–34]. Evidence that the RhoA/ROK pathway contributes to the vasoconstrictor component of pulmonary hypertension is demonstrated by effects of ROK inhibitors to acutely reduce pulmonary arterial pressure in both chronically hypoxia rats and in the hypoxia/SU5416 rat model of angioproliferative PAH [32, 33, 35, 36]. Findings from the current study directly demonstrate that RhoA/ROK contribute to both the enhanced basal vascular tone and agonist-induced vasoreactivity in hypoxia/SU5416-treated rats. Moreover, vasoconstrictor reactivity to ET-1 in small pulmonary arteries is greater in hypoxia/SU5416-treated rats than in rats exposed to chronic hypoxia alone. This is a novel finding since the more severe pulmonary hypertension accompanying the hypoxia/SU5416 model has been largely attributed to the development of occlusive neointimal and fibrotic plexiform lesions. Interestingly, small pulmonary arteries from hypoxia/SU5416 rats exhibited a modest reduction in medial cross-sectional area of SMA-positive immunofluorescence vs. chronic hypoxia alone, rather than the expected increase. Although this finding could be interpreted as reduced remodeling, we construed this as a likely indication of smooth muscle de-differentiation. The medial layer of arteries from hypoxia/SU55416 rats was often fragmented (Figs 4–6) with more de-differentiated smooth muscles cells (myofibroblasts) that express lower levels of SMA and higher levels of vimentin (marker of mesenchymal cells). These de-differentiated smooth muscle are known to have much higher rates of proliferation and migration [37] and were a central feature in the observed formation of arterial lesions in these animals. Taken together, the current findings suggest both the formation of vascular lesions and enhanced ROK-mediated vasoconstrictor responsiveness contribute to the severity of angioproliferative PAH.

Oxidative stress associated with pulmonary hypertension is thought to result from an imbalance of oxidant production and antioxidant capacity. Evidence for a vascular source of ROS in the hypertensive pulmonary circulation comes from studies in our laboratory that have identified an effect of chronic hypoxia to increase ROS levels in pressurized small pulmonary arteries as assessed by the O_2_^-^ -sensitive indicator dihydroethidium [8, 9]. Consistent with these findings are studies using an array of superoxide detection methods that have revealed increases in lung superoxide levels in chronically hypoxic mice [38, 39]. NADPH oxidase isoforms [38–41], xanthine oxidase [42], and mitochondria [43, 44] are major sources of ROS implicated in the development of pulmonary hypertension. In addition to increased superoxide generation, various models of pulmonary hypertension and patients with idiopathic PAH display decreased antioxidant capacity [10–17]. For example, SOD1 and SOD3 expression and activity are decreased in chronic hypoxia-induced pulmonary hypertensive mice, rats, calves, and piglets [12, 13, 15, 45, 46] and in a lamb model of persistent pulmonary hypertension of the newborn [47]. Furthermore, fawn-hooded rats, which have an epigenetic silencing of SOD2 expression/activity, and SOD1 knockout mice develop spontaneous pulmonary hypertension [10, 11, 14]. These animal studies further correlate with evidence of significantly lower SOD mRNA and SOD activity in patients with idiopathic PAH compared to healthy individuals [16, 17]. Interestingly, SOD-1 immunoreactivity is markedly absent in neointimal lesions of hypoxia/SU5416 rats [18], suggesting a potential role for oxidative stress in the development of angioproliferative PAH.

Previous studies provide evidence that TEMPOL normalizes RVSP and reduces right ventricular hypertrophy in chronically hypoxic rats [48], thus demonstrating a major contribution of ROS to the development of hypoxia-associated pulmonary hypertension. Similarly, the antioxidant, N-acetylcysteine (NAC) attenuates both chronic hypoxia- and monocrotaline-induced pulmonary hypertension [42, 49, 50], and overexpression of SOD reduces pulmonary hypertension resulting from chronic hypoxia [45], monocrotaline [51], and in lambs with prenatal ligation of the ductus arteriosus [52]. In contrast, the dietary supplement Protandim, which increases expression of antioxidant enzymes such as SOD and heme-oxygenase-1, neither reduced RVSP nor pulmonary vascular remodeling in chronic hypoxia/SU5416-treated rats, but prevented right ventricular hypertrophy and preserved right ventricular function [18]. While such findings support a protective effect of antioxidant strategies in treatment of right heart failure associated with severe PAH, the therapeutic efficacy of antioxidant strategies and the relative contributions of ROS to the vasoconstrictor and arterial remodeling components of angioproliferative PAH are largely unknown.

We have previously identified a central role for ROS in coupling both receptor stimulation and membrane depolarization to RhoA/ROK activation in small pulmonary arteries from chronically hypoxic rats [8, 9]. Consequently, a major focus of the present study was to evaluate whether ROS contribute to enhanced basal vascular tone, agonist-induced vasoreactivity, and the development of PAH in hypoxia/SU5416-treated rats. In agreement with this possibility, we found that pulmonary arterial smooth muscle cells from both chronically hypoxic and hypoxia/SU5416-treated rats exhibited greater superoxide levels compared to control animals. Scavenging superoxide with TEMPOL also decreased basal tone development and normalized vasoconstrictor sensitivity to ET-1 in each group to the level of normoxic controls, similar to that observed following ROK inhibition. In addition, TEMPOL attenuated hypoxia/SU5416-induced increases in RVSP, but did not normalize RVSP to control levels, as seen with animals exposed to chronic hypoxia alone. These findings are consistent with a contribution of ROS to the development of angioproliferative PAH in part through induction of spontaneous pulmonary arterial tone and greater sensitivity to vasoconstrictor stimuli.

Despite the reduction of RVSP, superoxide generation, and vascular reactivity, chronic TEMPOL treatment unexpectedly failed to reduce the hypoxia-induced pulmonary arterial muscularization and right heart hypertrophy. Rather, scavenging of ROS in hypoxia/SU5416-treated rats caused an unexpected increase in arterial muscularization, vimentin, and HSP-47 expression, and severity of adventitial fibrotic lesions. The mechanism by which TEMPOL exacerbated the arterial remodeling response to hypoxia/SU5416, however, is not clear. ROS are essential signaling molecules that are tightly regulated to maintain physiological homeostasis. A biphasic effect of ROS has been demonstrated on cellular proliferation in which low levels (submicromolar) actually induce growth but higher concentrations induce apoptosis [53]. It is possible that TEMPOL decreases superoxide levels thereby disrupting oxidative regulation of proliferation and host defense resulting in excessive proliferation and fibroblast activation. Therefore, the effect of TEMPOL to lower RVSP in both the chronic hypoxia- and hypoxia/SU5416-treated rats appears to be largely mediated by a reduction in vasoconstrictor reactivity, despite a paradoxical increase in arterial remodeling and fibrosis.

Although O_2_^·−^ is generally associated with contraction of pulmonary arteries, both contraction and relaxation have been observed in response to H_2_O_2_ [54–57]. As a consequence of reduced SOD expression and activity, a decrease in H_2_O_2_ levels has also been reported following chronic hypoxia exposure [11, 12, 14, 15, 58], as well as in other experimental models of spontaneous PH and in humans with idiopathic PAH [10, 11, 14]. This decrease in H_2_O_2_ is thought to contribute to proproliferative and antiapoptotic effects that are, in part, mediated by hypoxia-inducible factor 1α (HIF-1α) and involve a decrease in K_v_1.5 channel expression and elevated [Ca^2+^]_*i*_ levels [11]. In contrast to these reports, other laboratories show that H_2_O_2_ stimulates cell migration, proliferation, and differentiation in the pulmonary circulation [59–61]. It is therefore possible that TEMPOL, as an SOD mimetic, generates an abundance of H_2_O_2_ leading to excessive proliferation and vasodilation. However, based on our current findings this seems unlikely since TEMPOL induction of H_2_O_2_-specific oxidative stress was independent of chronic hypoxia or SU5416.

In summary, our data show that increasing antioxidant capacity through administration of the SOD mimetic, TEMPOL, reduced RVSP and vasoconstrictor reactivity in both chronic hypoxia and hypoxia/SU5416 treated rats. However, this response to TEMPOL was associated with an unexpected increase in medial hypertrophy and adventitial fibrotic lesion formation in hypoxia/SU5416 rats. The reason TEMPOL only increased the severity of arterial remodeling in the hypoxia/SU5416- and not chronic hypoxia-treated rats is not clear, but may result from phenotypic changes in cellular responsiveness to ROS in angioproliferative PAH. Together these data suggest that, despite protective effects of TEMPOL in limiting vasoconstrictor reactivity and the increase in RVSP, the exacerbation of arterial remodeling may pose therapeutic limitations of using an SOD mimetic in severe PAH.
